# Fe_3_O_4_/BC for Methylene Blue Removal from Water: Optimization, Thermodynamic, Isotherm, and Kinetic Studies

**DOI:** 10.3390/ma18092049

**Published:** 2025-04-30

**Authors:** Sharf Ilahi Siddiqui, Naha Meslet Alsebaii, Azza A. Al-Ghamdi, Reema H. Aldahiri, Elham A. Alzahrani, Sumbul Hafeez, Seungdae Oh, Saif Ali Chaudhry

**Affiliations:** 1 Environmental Chemistry Research Laboratory, Department of Chemistry, Jamia Millia Islamia, New Delhi 110025, India; sharfillahi@ramjas.du.ac.in; 2 Department of Chemistry, Ramjas College, University of Delhi, New Delhi 110007, India; 3 Department of Chemistry, Faculty of Science, King Abdulaziz University, P.O. Box 80200, Jeddah 21589, Saudi Arabia; nalsebaii@kau.edu.sa; 4 Department of Chemistry, College of Science, University of Jeddah, Jeddah 21959, Saudi Arabia; aaalgamdi@uj.edu.sa (A.A.A.-G.); rhal-dhahery@uj.edu.sa (R.H.A.); 5 Department of Chemistry, College of Science, University of Ha’il, Ha’il 81451, Saudi Arabia; elh.alzahrani@uoh.edu.sa; 6 Department of Civil and Environmental Engineering, Villanova University, Villanova, PA 19085, USA; sumbulhafeez03@gmail.com; 7 Department of Civil Engineering, College of Engineering, Kyung Hee University, Yongin 17104, Republic of Korea

**Keywords:** black cumin (BC) carbon framework, Fe_3_O_4_/BC magnetic biosorbent, wastewater treatment, kinetic/isotherm analyses, economic analysis

## Abstract

In this research, a nanoscale magnetic biosorbent was synthesized by incorporating magnetic nanoparticles (Fe_3_O_4_ NPs) into a natural carbon framework derived from black cumin (BC) seeds. The prepared Fe_3_O_4_/BC was utilized as a low-cost, eco-friendly, and reusable nanobiosorbent for the removal of organic (e.g., methylene blue (MB) dye) pollutants from synthetic solutions. The results indicated that Fe_3_O_4_/BC had extensive surface oxygenous functional groups with a high affinity for MB dye capture at different concentrations such as 10–60 mg L^−1^. The optimization results suggested the removal of ~99% of methylene blue from its initial concentration (i.e., 10 mg L^−1^) using 2.0 g L^−1^ of Fe_3_O_4_/BC at pH = 7, temperature = 27 °C, and contact time = 120 min, with equilibrium adsorption capacity = 5.0 mg g^−1^ and partition coefficient = ~57.0 L g^−1^. The equilibrium adsorption efficacy at the highest initial concentration (i.e., 60.0 mg L^−1^) was found to be 29.0 mg g^−1^. The adsorption isotherm was well explained by the Freundlich model for MB. The renderability of this magnetic bioadsorbent by acid treatments showed a ~66% decline in removal efficiency (%) (~99% to ~33%; ~5.0 to ~1.7 mg g^−1^) for MB after six repetitive cycles of adsorption and desorption. The current Fe_3_O_4_/BC gives a better partition coefficient than previously reported acid-washed BC seeds and other BC-seed-based nanobioadsorbents, Hence, a synthesized Fe_3_O_4_/BC nanobiosorbent demonstrates potential for use in treating water contaminated with organic pollutants.

## 1. Introduction

Nanomaterials (NMs) have been intensively implemented to help eliminate chemicals in various environmental media via the adsorption treatment process [1,2]. Currently, a magnetic sorbent is one of the preferable techniques in the existing advanced research on adsorption-based nanotechnology for the enhanced recovery of powdered nanosorbents from aqueous solutions under the impacts of an external magnetic field [3,4]. Specifically, many magnetite (Fe_3_O_4_)-based nanocomposites have been explored for their effectiveness in selectively removing a variety of dyes such as methylene blue (MB) and Congo red from aqueous solutions [5]. Their effectiveness is attributed to their cost-effective production and non-toxic nature [6,7,8].

Nonetheless, producing most of these magnetic nanocomposite sorbents involves the utilization of activated carbon and/or biochar supports for loading magnetic nanoparticles (NPs) [8,9]. Notably, synthesizing activated carbon/biochar from biowaste products involves chemical treatments and consumes a significant amount of energy, which increases the costs of production and poses the risk of secondary pollution throughout the synthesis process [10,11]. Additionally, it has been observed that most NPs non-covalently adhere to activated carbon/biochar nanocomposites (since their surfaces do not have functional groups). This leads to the rapid leaching of NPs into water throughout the adsorption process [12]. Continuously aggregating these discharged NPs poses significant risks to marine life as well as human health [12].

The aforementioned limitations might be mitigated via searching for a new carbon support and enhancing the interaction between magnetic NPs and the carbon support. In this respect, natural plant materials with enormous oxygen/amine functionalities were used effectively for the synthesis of nanobiocomposites, such as magnetic Azolla and fig leaves [13], Fe_3_O_4_-GLP@ CAB [14], magnetic date seeds [15], and magnetic Forsythia suspensa leaves [16].

In this respect, the seeds of cultivated BC are natural and versatile carbon frameworks with a number of hydroxyl (-OH) and carboxylic (-COOH) groups at the surface. The possible significance of BC particles as biosorbents has already been demonstrated in several prior studies [17,18]. For example, an oxygenated functionalized BC seed surface (e.g., -OH and -COOH) could accelerate the adsorption of cationic pollutants such as cationic dyes and heavy metals in wastewater solutions [17,18]. These oxygenated functional groups could serve as scaffolds for the synthesis and development of metal/oxide NPs by the accommodation of metal (M^n+^) ions [19,20]. Given this potential, a great deal of work has gone into creating hybrid nanosorbent BC particles that interact with various NMs, e.g., reduced graphene oxide/zirconium oxide/BC-based hybrid composites (rGO-BC@ZrO_2_) [19], BC with Fe_2_O_3_-ZrO_2_ particles (Fe_2_O_3_-ZrO_2_/BC) [20], and BC with Fe_2_O_3_-SnO_2_ particles (Fe_2_O_3_-SnO_2_/BC) [21]. However, to our knowledge, all these reported BC-based nanocomposite sorbents lack magnetic behavior for easy recovery and separation from aqueous solutions. Hence, in this work, we propose hybridization between BC and iron oxide NPs (e.g., Fe_3_O_4_ NPs with magnetic features [14]) to design nanobiomagnetic Fe_3_O_4_/BC nanocomposite sorbents for wastewater treatment.

Recently, a group of researchers [22] magnetized BC seeds and investigated their antifungal activity; however, no studies can be found that use magnetized BC for water treatment. Based on the expected advantages, the objective of this research work was to design a smart Fe_3_O_4_/BC nanobiosorbent for methylene blue (MB) removal in water solutions. To the best of our knowledge, the use of Fe_3_O_4_/BC as a magnetic nanobiosorbent for organic pollutant removal is reported in this work for the first time.

## 2. Experimental Procedures

### 2.1. Reagents and Materials

Anhydrous ferric chloride (FeCl_3_; purity 96.0%) and ferrous sulfate heptahydrate (FeSO_4_.7H_2_O; purity 99.0%) were supplied by Sigma-Aldrich (Darmstadt, Germany). Sodium hydroxide (NaOH; purity 97.0%), hydrochloric acid (HCl; assay purity 37%), sulfuric acid (H_2_SO_4_; MW 98.08; purity 98.0%), and methylene blue (MB; purity 97.0% and λmax at 660 nm) were purchased from Sigma-Aldrich (Darmstadt, Germany). Potassium nitrate (KNO_3_; purity 99.0%) was procured from Sigma-Aldrich (Darmstadt, Germany). The BC was collected from New Delhi, India.

### 2.2. Preparation of Fe_3_O_4_/BC

The preparation of nanoscale magnetic Fe_3_O_4_/BC bioadsorbent was carried out, following the simple co-precipitation method given in the literature [22]. In detail, the BC was rinsed with double distilled water, followed by drying overnight at 80–90 °C, ground to powder, and finally sieved through 60–200 mesh. One gram of sieved BC was suspended in 100 mL distilled water under ultrasonication (20 kHz) for 5 min at 27 ± 1 °C. Afterwards, 50 mL solutions containing 0.1M FeCl_3_ and 0.05M FeSO_4_ were introduced to BC suspension. The residual solutions were stirred at 950 rpm and 60 ± 5 °C for 30 min. After 30 min, the pH of the solution was maintained at pH 10.5 ± 1 by adding 8.0 M NaOH solution dropwise in atmospheric conditions and followed by further stirring for 15 min under the same conditions. Finally, the solution was cooled down to room temperature and the resulting precipitate-like particles settled. The resulting precipitate was filtered and this was followed by multiple washings with distilled water. The washed Fe_3_O_4_/BC composite was obtained by utilizing an exterior magnetic field at 60 °C for 48 h in a vacuum oven.

In the current research, the BC functionalized with a cellulosic surface enriched with numerous functional groups such as -COOH and -OH served as a carbon framework to embed Fe^2+^/Fe^3+^ ions into the BC surface. The Fe^2+^/Fe^3+^ ions undergo oxidation via co-precipitation to produce Fe_3_O_4_ NPs under the BC carbon framework upon the addition of NaOH. Note that all materials utilized in the current study were of analytical grade, as presented in Section 2.1. The prepared Fe_3_O_4_/BC was also characterized by various techniques (given in Section 2.3) to understand its surface chemistry, crystallinity, morphology, magnetism, and texture properties.

### 2.3. Instrumentation

Fourier transform infrared (FTIR) spectra were collected with a Nicolet 6700 FTIR spectrometer, Waltham, Massachusetts, USA after the formation of pallets in KBr with the resolution range of 4000–400 cm^−1^. The X-ray diffraction (XRD) pattern was demonstrated (PW-3710 diffractometer, Philips, Cambridge, MA, USA) using Cu-Kα radiation (λ = 1.54 Å) and a Cu filter, 35 kV, 30 mA. A scanning electron microscope (SEM; Zeiss EVO 18 SEM, Jena, Germany) equipped with EDAX and a transmission electron microscopy (TEM) using a Technai G2 20, Hillsboro, Oregon, USA were used to analyze the structure and morphology of the constructed products. The magnetic behavior of Fe_3_O_4_/BC was confirmed by monitoring at 27 °C in a magnetic field (between 1.5 and −1.5 Tesla) by employing an MPMS-XL-7 magnetometer (MPMS-XL-7 magnetometer, Quantum Design, San Diego, CA, USA). The degree of carbon framework degradation and heat stability were determined using these profiles.

### 2.4. Zero-Point Charge (pHzpc) of the Fe_3_O_4_/BC

The Fe_3_O_4_/BC pHzpc was demonstrated using the salt addition method [23]. Briefly, a series of 100 mL Erlenmeyer flasks were filled with 0.2 g of Fe_3_O_4_/BC that had been dispersed in 50 mL of 0.01M potassium nitrate (KNO_3_) solutions with a starting pH value of 2–10. The pH of solutions was adjusted by using 0.1M HCl and NaOH solutions. Afterwards, all flasks were subjected to vigorous agitation in an incubator at 215 rpm and 27 ± 1 °C for 2 h, then left for 24 h of equilibration under static conditions. Finally, the pHzpc value of Fe_3_O_4_/BC was determined from the plot between initial pH and its change (∆pH = initial − final pH) after 24 h of equilibration.

### 2.5. Preparation of Adsorbate (MB) Solutions

One gram of MB was dissolved in one liter of double-distilled water to provide a stock aqueous solution with a concentration of 1000 mg L^−1^. During the adsorption experimental investigations, the generated stock solutions of MB dye were then diluted to the recommended quantities. The concentration of MB was measured at a λ_max_ value of 660 nm using a spectrophotometer.

### 2.6. Adsorption Experiments

The adsorption capability of Fe_3_O_4_/BC nanobiosorbent for MB dye removal was tested with a batch adsorption technique. Herein, all adsorption experiments have been performed by the addition of 0.02 g of Fe_3_O_4_/BC and 10 mL MB solution in a sequence of 50 mL Erlenmeyer flasks, then agitated at 215 rpm and 27 ± 1 °C. Using the one variable at a time (OVAT) optimization method, the adsorption performance of Fe_3_O_4_/BC for target pollutants was investigated and optimized in relation to a list of key variables controlling the adsorption processes. The tested variables were as follows: the amount of Fe_3_O_4_/BC (1.0–5.0 g L^−1^), pollutant concentrations (MB (10–60 mg L^−1^)), temperatures (27, 35, and 45 °C), solution’s pH (2.0–10.0), and reaction contact time (15–120 min). After each adsorption experiment, the Fe_3_O_4_/BC nanocomposite was excluded from the prepared solution by utilizing an external magnet. Finally, the residual MB concentration present in the treated solutions was determined at the λ_max_ value of 660 nm, utilizing a UV-Vis spectrophotometer (T80-UV/VIS, PG instruments Ltd., Leicestershire, UK).

The adsorption capacity (*Q_e_* (mg g^−1^), Equation (1)) of Fe_3_O_4_/BC nanocomposite for MB and the removal percentage (Equation (2)) were computed using the following formulas [18].

(1) $$Adsorption\;capacity\;at\;equilibrium\;(Q_{e})=\left(C_{o}-C_{e}\right)v/m$$

(2) $$Removal\;efficiency\;\left(\%\right)=\left(C_{o}-\frac{C_{e}}{C_{o}}\right)100$$

where *Q_e_* (mg g^−1^) is expressed as equilibrium adsorption capacity. The starting and equilibrium MB concentrations (mg L^−1^) are *C_o_* and *C_e_*, respectively. The Fe_3_O_4_/BC nanocomposite mass is *m* (g) and the solution volume is *V* (L).

### 2.7. Thermodynamic Analyses for MB Adsorption

In terms of adsorption thermodynamics (27–45 °C), the change in entropy (Δ*S°*: kJ mol^−1^ K^−1^), enthalpy *(*Δ*H°*: kJ mol^−1^), and Gibbs free energy (Δ*G°*: kJ mol^−1^) for the transfers of MB molecules from liquid to solid adsorbent phases were computed as a function of their concentrations (see Section 2.2) at equilibrium contact time (120 min). The thermodynamic parameters were estimated using the following equation [24].

(3) $$∆G{}^{\circ}=-RTlnK_{d}$$

*R* expressed as the universal gas constant (8.314 J K^−1^ mol^−1^), *K_d_ = Q_e_/C_e_* is the distribution coefficient, and *T* is the absolute temperature (K).

Once the ∆*G°* values have been computed, Formula (4) may be used to evaluate the Δ*H°* and Δ*S°*, respectively.

(4) ∆G°=∆H°−T∆S°

### 2.8. Isotherm Studies for MB Adsorption

The Langmuir, Freundlich, and Temkin isotherm models were used in this study to simulate the relationship between adsorbed and residual concentrations of MB molecules onto Fe_3_O_4_/BC nanocomposite at equilibrium [18]. The Langmuir model (Equation (5)) is useful to describe the monolayer adsorption [25].

(5) $$\frac{C_{e}}{Q_{e}}=\frac{C_{e}}{Q_{o}}+\frac{1}{Q_{o}b}$$

(6) $$R_{L}=\frac{1}{\left(1+bC_{e}\right)}$$

where *Q_o_* (mg g^−1^) represents the adsorbent maximal monolayer capacity and *b* (L g^−1^) is the Langmuir constant. Based on the Langmuir constant, *R_L_* (the separation constant) is calculated (Equation (6)) to verify the adsorption nature [26].

The multilayer sorption onto the homogeneous/heterogeneous solid surface was explained by the Freundlich isotherm (Equation (7)) [27].

(7) $$logQ_{e}=logK_{F}+\frac{1}{n}logC_{e}\;$$

Freundlich constants denoted by *k_F_* ([mg g^−1^][L mg^−1^]^1/n^) and *n* signify adsorption capacity at unit concentration and intensity of adsorption, respectively.

The Temkin isotherm (Equation (8)) is applicable to examine the indirect solute–solid interaction during the adsorption process. It assumed that the surface sorbent coverage with solute molecules increases as heat of adsorption *(b_T_)* reduces [28].

(8) $$Q_{e}=\frac{RT}{b_{T}}lnA_{T}+\frac{RT}{b_{T}}lnC_{e}$$

where the constants for the maximal binding energy and heat adsorption are *A_T_* (L g^−1^) and *b_T_* (kJ mol^−1^), respectively.

### 2.9. Kinetic Studies for MB Adsorption

In optimum operation conditions, the kinetic studies have been performed to demonstrate the adsorption mechanism for MB on Fe_3_O_4_/BC nanobiosorbent and predict the rate-controlling adsorption process. In this respect, three kinetic models of pseudo-first-order (PFOM, Equation (9)), pseudo-second-order (PSOM, Equation (10)), and Elovich (Equation (11)) equations were used to examine the physicochemical interaction between MB and Fe_3_O_4_/BC surfaces [18,29]. In addition, a mechanistic kinetic diffusion model (e.g., intra-particle diffusion (IPD), Equation (12)) has been implemented to determine the rate-controlling adsorption method [18,29].

(9) $$\log\left(Q_{e}-Q_{t}\right)=logQ_{e}-\frac{K_{1}}{2.303}t\;$$

(10) $$\frac{t}{Q_{t}}=\frac{1}{h}+t/Q_{e}$$

(11) $$Q_{t}=\frac{1}{\beta }\ln\left(\alpha \beta \right)+\frac{1}{\beta }lnt$$

The *k*_1_ (min^−1^) is expressed as the PFOM kinetic rate constant, *h* is the starting rate constant (*h = k_2_Q_e_*^2^), and *k*_2_ (mg^(1−n)^ L^n^ g^−1^) is PSOM’s overall kinetic rate constant. *Q_t_* (mg g^−1^) is solute removal capacity at time *t* (min). Additionally, Elovich constants *α* (mg g^−1^ min^−1^) and *β* (g mg^−1^ min^−1^) control the starting rates of adsorption and desorption, respectively. The *β* value also explains the degree of surface covering and chemical interaction activation energy [29].

The IPD (Equation (12)) model is expressed as follows.

(12) $$Q_{t}=K_{ipd}t^{0.5}+C$$

### 2.10. Adsorption Activation Energy

In order to understand the mechanism of adsorption, whether it is physical adsorption or chemical adsorption, it is important to find out the activation energy. The activation energy can be determined by the Arrhenius equation (Equation (13)) [26].

(13) $$lnK_{2}=lnA-\frac{Ea}{RT}$$

Herein, *K*_2_ is the rate constant at various temperatures (27–45 °C), *Ea* is the activation energy (J mol^−1^), *A* is the Arrhenius frequency factor, *R* is the molar gas constant (8.314 J mol^−1^ K^−1^), and *T* is the absolute temperature (K).

By plotting a plot between *lnK*_2_ and 1/*T*, we can find the value of *Ea*.

### 2.11. Recyclability of Fe_3_O_4_/BC

The regeneration method was performed using desorption of adsorbed MB on the Fe_3_O_4_/BC adsorbent surface using solutions of 0.1 M HCl. Herein, the exhausted Fe_3_O_4_/BC sorbent was dispersed in 100 mL of HCl solution, then agitated at 215 rpm and 27 ± 1 °C for 4 h. The regenerated Fe_3_O_4_/BC was thoroughly washed with distilled water until the solution of pH became neutral, followed by drying. The dried Fe_3_O_4_/BC was further used for the removal of MB pollutants using the adsorption method from water solutions in optimum conditions. This desorption/adsorption process was repeated over six cycles to assess the adsorption stability of Fe_3_O_4_/BC in the environmental field.

## 3. Results and Discussion

### 3.1. Characterization Data of Fe_3_O_4_/BC

Herein, Fe_3_O_4_/BC biocomposite (relative to the raw BC framework) was characterized by Fourier transform infrared (FTIR) spectra, powder X-ray diffraction (XRD), field-emission scanning electron microscope (FE-SEM), energy-dispersive X-ray (EDX), transition electron microscope (TEM), and vibrating sample magnetometer (VSM) analytical techniques. Notably, all characteristic data confirmed the successful impregnation of spherical and/or quasi-spherical NPs of Fe_3_O_4_ in a BC carbon framework, with strong binding between Fe_3_O_4_ and BC functionalities, to develop nanocomposite binding with high chemical/thermal stability and magnetic properties, as discussed below.

**Figure 1:**
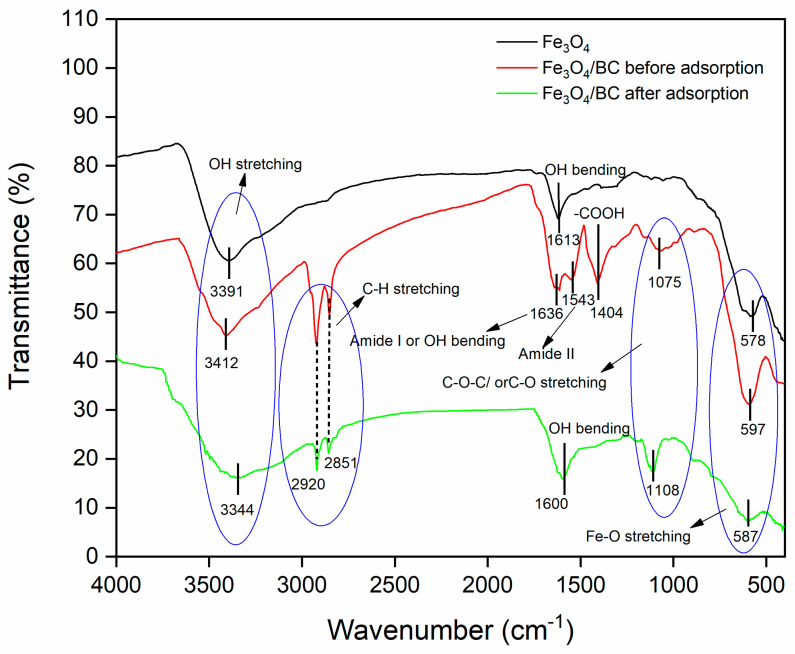
FTIR spectrum of Fe_3_O_4_ (black line), Fe_3_O_4_/BC before adsorption (red line), and Fe_3_O_4_/BC after adsorption (green line).

The FTIR spectrum of bare Fe_3_O_4_ NPs (prepared by a similar co-precipitation method) showed the vibration peaks of O-H (stretching at ~3391 cm^−1^ and bending at ~1613 cm^−1^; due to moisture absorbed) and metal–oxygen (Fe-O at ~578 cm^−1^) (Figure 1; black line). Compared to the FTIR spectrum of bare Fe_3_O_4_, the FTIR spectrum of Fe_3_O_4_/BC composite (Figure 1; red line) indicated the presence of various oxygen functionalities, which are related to the presence of the cellulosic framework of BC seed powder in the range of 600–4000 cm^−1^. In particular, the FTIR spectrum of Fe_3_O_4_/BC (Figure 1; red line) showed the following vibration peaks: O-H stretching (at ~3391 cm^−1^), stretching peaks (-C-H) of -CH_3_**/**-CH_2_ moieties (at ~2920 and 2851 cm^−1^) [30]), acidic carboxyl groups of cellulose (e.g., -COOH and C-O-C/C-O at ~1404 and ~1075 cm^−1^, respectively) [31,32,33], and amino acids of the protein–peptide bonds (e.g., N-H stretching amide II at ~1543 cm^−1^) [18,34]. The peak at ~1636 might be due to the peak of amide I and/or -OH bending [18,33,34]. All these peaks of functional groups observed in the FTIR spectrum of Fe_3_O_4_/BC showed strong agreement with the FTIR spectrum of BC seeds (with some shifting and disappearance) reported in our previous study (Figure S1). Furthermore, the newly observed stretching vibrational peak of Fe-BC (-O-Fe) at 597 cm^−1^ [35] indicates the successful impregnation of BC with Fe_3_O_4_ NPs and the presence of strong bonding among BC and Fe_3_O_4_. This is likely to suggest the bonding between the functional sites of BC (Figure S1) and Fe^3+/^Fe^2+^ during the in situ impregnation of Fe_3_O_4_ NPs. Similar FTIR results were observed for the previously reported magnetic Forsythia suspensa leaf [16] and quince seed mucilage nanocomposites [36].

**Figure 2:**
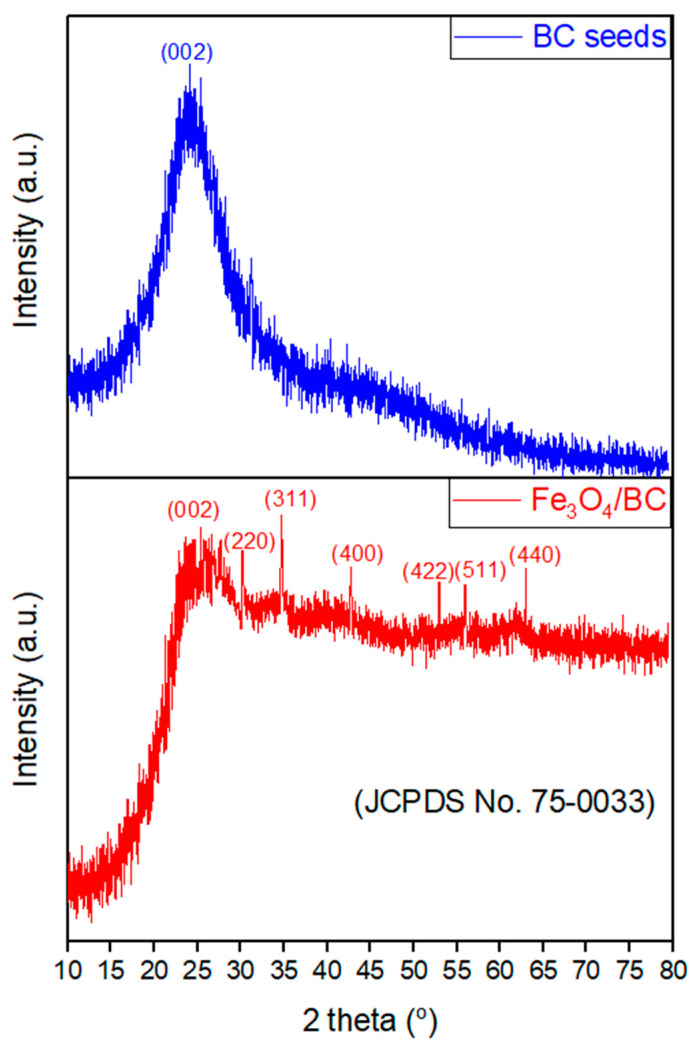
XRD analysis of BC, reprinted from our previous study with permission from Siddiqui and Chaudhry [39], Copyright (2018) Elsevier (License No. 5990980697967), and Fe_3_O_4_/BC.

The XRD pattern of Fe_3_O_4_/BC (Figure 2; red line) showed characteristic patterns of Fe_3_O_4_ crystals at 2θ°of 30.1°, 34.9°, 42.8°, 53.0°, 56.0°, and 63.1°, belonging to [220], [311], [400], [422], [511], and [440], respectively [37]. These peaks are well-matched with JCPDS card no. 19-0629 of Fe_3_O_4_ crystals [38]. A broad peak corresponding to the [002] plane is also observed in the XRD spectrum of Fe_3_O_4_/BC at ~25.0°, which represents the carbon surface due to the BC seeds (see the comparative study with XRD of BC (Figure 2; blue line; since this study is an extension of our previous study [39], the XRD of BC was used with copyright permission from the publisher). This reflects the interaction between the crystalline Fe_3_O_4_ and amorphous BC [26,37,40].

Nevertheless, it should be noticed that the color of the Fe_3_O_4_/BC sample changed from brownish-black to brown during the XRD study, which could have been caused by slight oxidation of Fe_3_O_4_ (magnetite) to the γ-Fe_2_O_3_ (maghemite) during preparation of composites through the investigated procedure [41]. This might suggest that the prepared Fe_3_O_4_/BC composite contains both Fe_3_O_4_ and γ-Fe_2_O_3_ phases. The Fe_3_O_4_ and γ-Fe_2_O_3_ showed exact XRD patterns so it is challenging to distinguish between these two phases [42]. Therefore, the predominate phase of the Fe_3_O_4_/BC composite might be determined using the XRD angular value as shown in Figure 2 (red line). In particular, the 2θ value of 35.35°, which belongs to the [311] plane, indicates the highest peak and confirms the presence of Fe_3_O_4_ (reported 2θ values for the [311] plane: 35.423° for Fe_3_O_4_ and 35.631° for γ-Fe_2_O_3_) [38]. Remarkably, the obtained 2θ value of the [311] plane of Fe_3_O_4_/BC is consistent (close) with the standard 2θ value for Fe_3_O_4_, confirming its dominant phase. Furthermore, the XRD peaks confirm that no other iron oxide is present in the prepared magnetic nanocomposite. Similar XRD results were observed for the previously reported quince seed mucilage magnetic nanocomposites [36].

**Figure 3:**
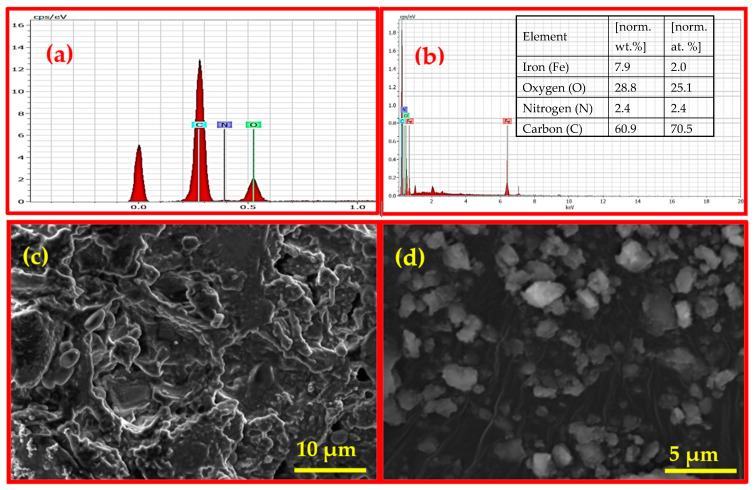
EDX analysis of (**a**) BC (reprinted from our previous study with permission from Siddiqui and Chaudhry [39], Copyright (2018) Elsevier (License No. 5990980697967)) and (**b**) Fe_3_O_4_/BC (inset: elemental composition) and SEM images of (**c**) BC (reprinted from our previous study with permission from Siddiqui and Chaudhry [20], Copyright (2019) Elsevier (License No. 5990980217818)) and (**d**) Fe_3_O_4_/BC.

**Figure 4:**
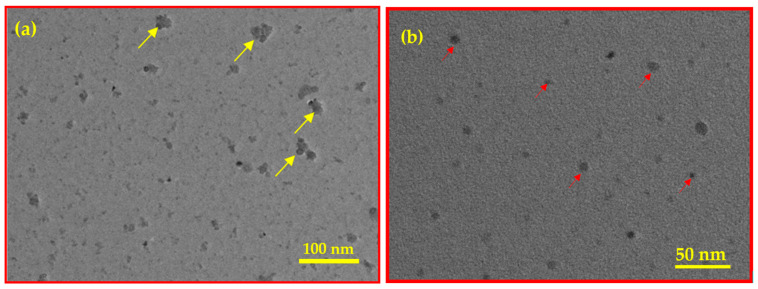
TEM images of BC (**a**) and Fe_3_O_4_/BC (**b**).

The structural appearance characteristic of Fe_3_O_4_/BC composite has been further demonstrated by using SEM, EDX, and TEM analysis, as shown in Figure 3 and Figure 4. The SEM image of Fe_3_O_4_/BC (Figure 3d) showed high surface roughness incorporating quasi-spherical-shaped bright particles (relative to the smooth surface of BC in Figure 3c; since this study is an extension of our previous study [20], the SEM image of BC was used with copyright permission from the publisher) due to the amalgamation of Fe_3_O_4_ within the BC carbon framework.

The identification of Fe_3_O_4_/BC due to the presence of C, N, O, and Fe elements with weight percentages of 60.87, 2.45, 28.81, and 7.87, respectively, was further supported by EDX elemental techniques (Figure 3b). The presence of C, N, and O in nanobiocomposite (Fe_3_O_4_/BC) is due to the carbon framework of BC seeds (Figure 3a; since this study is an extension of our previous study [39], the EDX of BC was used with copyright permission from the publisher) while Fe is present due to the NPs.

The TEM image of BC (Figure 4a) shows small black round substances belonging to the vacuoles (indicated with a yellow arrow), suggesting mature and electron-dense granules due to the cellulosic structure of BC. These vacuoles are represented as active sites of Fe_3_O_4_ NPs, with diameters ranging from 20–100 nm within the BC (see Figure 4b for Fe_3_O_4_/BC composite). The van der Waals collisions as well as the magnetic nature of NPs caused the production of aggregated Fe_3_O_4_ NPs (red arrow, Figure 4b) on the BC as seen by TEM analysis. This results in low dispersibility and stress on Fe_3_O_4_ on the BC surface, leading to a rough, non-uniform distribution of Fe_3_O_4_/BC.

**Figure 5:**
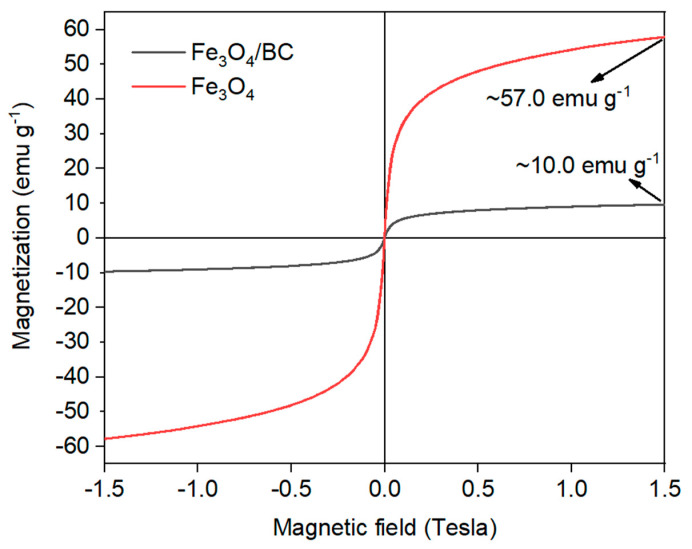
VSM of bare Fe_3_O_4_ (red line) and Fe_3_O_4_/BC (black line).

The magnetic behavior of Fe_3_O_4_/BC composite was also verified by VSM analysis at room temperature, as shown in Figure 5. As seen, the narrow hysteresis VSM loop of Fe_3_O_4_/BC with a very low coercive field and remanence value supported the magnetic significance of Fe_3_O_4_/BC at room temperature [40]. Nonetheless, the obtained saturation magnetism moment (Ms) of Fe_3_O_4_/BC composite was found to be approximately 10.0 emu g^−1^, that is reduced compared to bulk Fe_3_O_4_ (~90.0 emu g^−1^) listed in the previous works [43,44] as well as that prepared in this study as reference material (Figure 5; red line; ~57.0 emu g^−1^). This observation might be due to the partial oxidation of Fe_3_O_4_ to Fe_2_O_3_ and the presence of non-magnetic BC in the composite. Overall, the VSM results showed that Fe_3_O_4_/BC could be separated effectively after the adsorption process using a magnet.

### 3.2. Optimization of Fe_3_O_4_/BC Adsorption Behavior for MB Dye Pollutant

**Figure 6:**
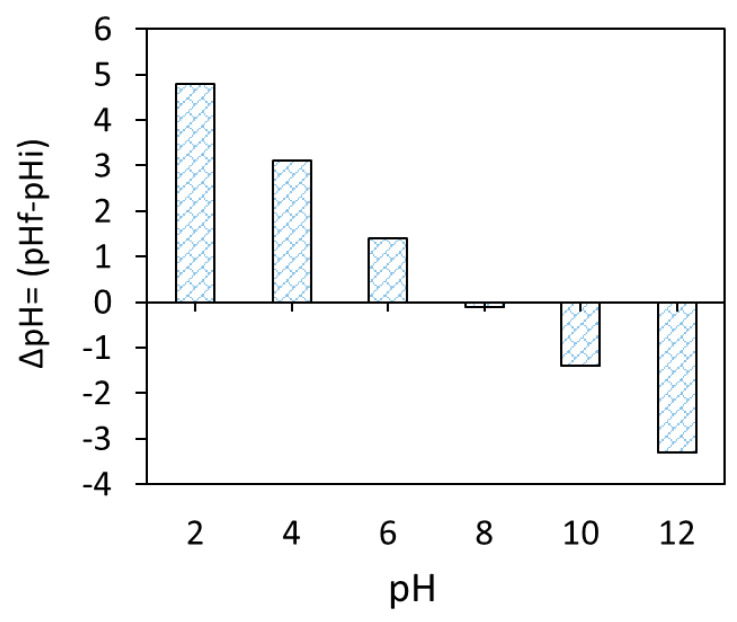
Zero-point charge of magnetic Fe_3_O_4_/BC.

Based on the obtained FTIR and pHzpc (pHzpc ≈ 8.0, Figure 6) data of the Fe_3_O_4_/BC surface, the adsorption of MB dye onto Fe_3_O_4_/BC can be controlled by electrostatic/non-electrostatic (H bonding) interaction through suitably adjusting solution pH values. Hence, in this work, the amount of MB dye adsorbed onto Fe_3_O_4_/BC adsorbent was further investigated and optimized as a function of four key variables (e.g., adsorbent dose, contact time, initial pollutant concentrations, temperature, and solution pH).

#### 3.2.1. Effect of Fe_3_O_4_/BC Dosage

**Figure 7:**
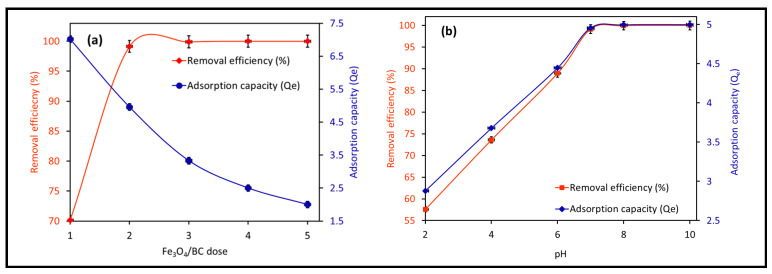
Effect of operation conditions on the adsorption of MB on magnetic Fe_3_O_4_/BC adsorbent: (**a**) effect of adsorption dose (at pH = 7, MB initial concentration = 10 mg L^−1^, contact time = 120 min, and temperature 27 °C) and (**b**) effect of solution pH (at adsorption dose = 2.0 g L^−1^, MB initial concentration = 10 mg L^−1^, contact time = 120 min, and temperature 27 °C) on MB adsorption capacity/removal efficiency (%) of magnetic Fe_3_O_4_/BC.

Figure 7a shows the effect of Fe_3_O_4_/BC dosage (1.0 to 5.0 g L^−1^) on the adsorption of 10 mg L^−1^ MB at fixed conditions of 120 min contact time, 27 ± 1 °C, and pH 7. The Fe_3_O_4_/BC adsorption capacity lowered from ~7.0 to 2.0 mg g^−1^ (for MB) by increasing nanobiosorbent dose from 1.0 to 5.0 g L^−1^ (Figure 7a). On the other hand, it was noted that approximately 99% of MB could be removed by increasing the Fe_3_O_4_/BC dose from 1.0 to 2.0 g L^−1^, followed by steady-state equilibrium curves (Figure 7a). The enhanced removal percentage with increasing Fe_3_O_4_/BC dose up to 2.0 g L^−1^ (as optimum value) may have been caused by the increased adsorption sites and stimulated collision between MB and Fe_3_O_4_/BC [20,29,45]. In contrast, the low adsorption capacity at a high adsorbent dosage (5 g L^−1^) could be a factor for three reasons: (i) the inversely proportional relationship of adsorption capacity to the adsorbent dose (see Equation (1)), (ii) the high capability of Fe_3_O_4_/BC for complete adsorption of M (≥99% removal) at a low dosage (2.0 g L^−1^); thus the increase in free available surface site numbers at a high dose could lead to their partial utilization (relative to lower Fe_3_O_4_/BC dosage (1.0 g L^−1^) with saturated adsorption sites), and/or (iii) the possible agglomeration of magnetic Fe_3_O_4_/BC composites at higher dosages may hinder the diffusion path of free MB adsorbate to the free active adsorption sites [46].

#### 3.2.2. Effect of Solution pH

The impact of pH changes from 2–10 on Fe_3_O_4_/BC adsorption efficacy for 10.0 mg L^−1^ MB in fixed conditions (27 ± 1°C and contact time of 120 min) is represented in Figure 7b at its optimum dose of 2.0-g L^−1^ Fe_3_O_4_/BC. The adsorption performance of Fe_3_O_4_/BC for both adsorbates is pH-dependent, with a maximum adsorption capacity at pH of 7.0–10.0 for MB. This pH–adsorption dependence can be explained based on the pHzpc value of Fe_3_O_4_/BC and the ionization/dissociation constant of MB vs. solution pH. At pH > pHzpc of 8, deprotonation of composite surface sites took place, leading negatively charged adsorbent surfaces (-O^−^ and COO^−^). The opposite trend can be found at acidic pH (i.e., pH < pHzpc: positively charged surface -OH_2_^+^ and COOH_2_^+^ created by the hydration process).

**Figure 8:**
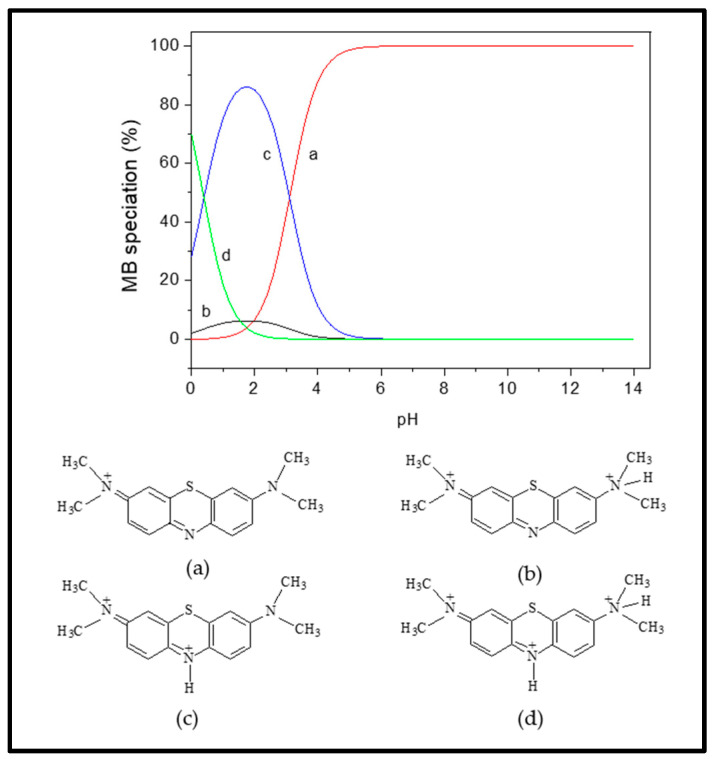
Distribution of MB species (**a**–**d**) at different pHs. Adapted from the Sousa et al. [46], with copyright permission, Elsevier, 2019.

**Figure 9:**
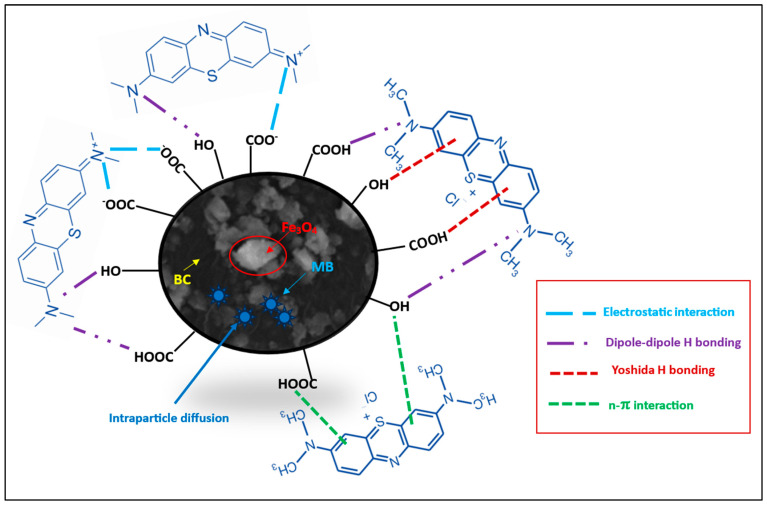
Proposed mechanism of adsorption of MB onto Fe_3_O_4_/BC.

Considering the MB pKa value (i.e., 3.8), the predominant forms of MB in solutions should be the cationic species at 4.0 < pH < 4.0 (Figure 8; species a–d) [46]. At lower solution pH (2.0 to 7.0) < pHzpc, the cationic forms of MB^+^ molecules (Figure 8) can only be attracted to the protonated Fe_3_O_4_/BC surface through non-electrostatic interactions, such as van der Waals interactions, hydrophobic interactions, and/or ion exchange between -OH_2_^+^ and COOH_2_^+^ (on the Fe_3_O_4_/BC surface) and MB^+^ molecules (deprotonation effect). In the alkaline medium (pH ≥ pHzpc), the induced electrostatic interaction mechanism between the negatively charged Fe_3_O_4_/BC (Fe_3_O_4_/BC-O^−^) surfaces and cation MB^+^ molecules (cationic species ‘a’ in Figure 8) would accelerate the adsorption process to achieve the maximum capacity for MB dye at pHs of 7 to 10. Schematic representation for these interaction mechanisms between MB^+^ molecules and the Fe_3_O_4_/BC surface across varying solution pHs is visualized in Figure 9, as verified by FTIR analysis (before and after adsorption) in Section 3.1.

#### 3.2.3. Effect of Adsorbate Concentrations and Temperature

**Figure 10:**
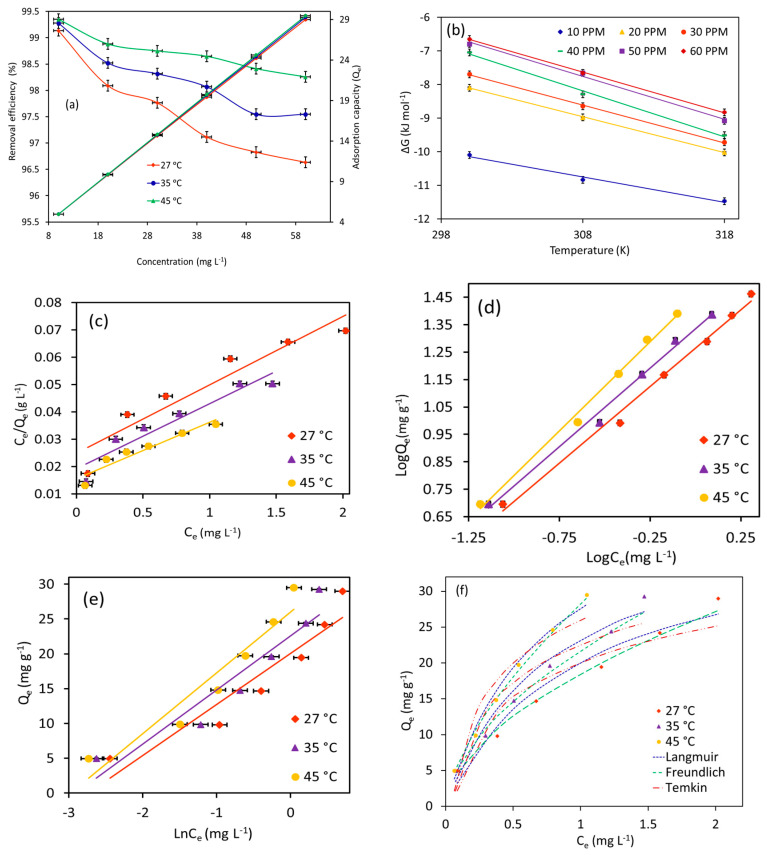
Plots of (**a**) effect of temperatures and initial concentrations on MB adsorption, (**b**) thermodynamics, (**c**) Langmuir isotherm, (**d**) Freundlich isotherm, (**e**) Temkin isotherm, and (**f**) isotherm simulation at different temperatures for MB adsorption onto magnetic Fe_3_O_4_/BC (at pH = 7, adsorption dose = 2.0 g L^−1^, MB initial concentration = 10–60 mg L^−1^, and contact time = 120 min).

The combinatorial effects of MB starting concentrations ranging from 10–60 mg L^−1^ and temperature ranging from 27–45 °C on adsorption performance (equilibrium adsorption capacity and removal efficiency (%)) of Fe_3_O_4_/BC (2.0 g L^−1^) were also tested, as shown in Figure 10a (in fixed conditions of 120 min contact time and pH 7). In Figure 10a, the adsorption uptake of Fe_3_O_4_/BC for MB molecules increases from ~5.0 mg g^−1^ to ~29.0 mg g^−1^ by increasing the starting concentration from 10 to 60 mg L^−1^. On the other hand, it should be noted that the removal efficiency (%) was decreased with the increase in the initial MB concentration (Figure 10a). The improved adsorption uptake at higher initial concentrations could probably be attributed to the extent of the driving force of MB concentration gradients that promoted the interactions of MB molecules with available surface sites [29,47,48,49]. Nevertheless, it is important to note that the binding sites with a fixed amount of Fe_3_O_4_/BC may achieve a saturation point with an increase in initial loading level of adsorbate concentrations, leading to reducing the removal percentage of tested pollutants (Figure 10) as compared to the increased adsorption capacity.

For the temperature effect, it was noted that increasing the temperature from 27 to 45 °C shows a slightly enhanced adsorption performance of Fe_3_O_4_/BC for MB uptake at all tested initial concentrations (Figure 10a). The favorable absorbent–adsorbate interaction with increased temperature indicates an endothermic and/or activated adsorption process [50]. With an increase in temperature, the kinetic energy and Brownian movement of adsorbate molecules in water solution could increase, leading to promoting the diffusion phenomenon of adsorbates to the Fe_3_O_4_/BC surfaces at higher temperatures [51]. Nonetheless, an increase in adsorbate kinetic energy at higher temperatures may lead to the breaking of physical interactions between MB adsorbate and Fe_3_O_4_/BC adsorbent surface (e.g., intermolecular H-bond, π–π interaction, and van der Waals bond), which play crucial roles in the adsorption process as discussed above [52]. This could slightly reduce the synergistic temperature effect on enhanced adsorption methods of Fe_3_O_4_/BC, especially at low starting concentrations of the pollutant (Figure 10a).

### 3.3. Thermodynamics of MB Adsorption

The thermodynamic variables, such as ΔH° (kJ mol^−1^), ΔS° (kJ mol^−1^ K^−1^), and ΔG° (kJ mol^−1^), for adsorption of MB over the entire range of concentrations of MB (10 to 60 mg L^−1^) are listed in Table 1. Notably, the values of positive ΔH° represented favorable endothermic adsorption in nature for MB onto Fe_3_O_4_/BC [24]. The positive ΔS° revealed increased disorder at the solid–liquid interface due to the change in hydration conditions of the adsorbed dye and/or Fe_3_O_4_/BC throughout the adsorption method [24]. The positive ΔS° values also indicate the redistribution of energy (rotational and translational) and randomness between the analytes and the Fe_3_O_4_/BC surface sites during the process of adsorption. This indicates that the favorable endothermic adsorption process can be promoted by activating the desolvation process of the adsorbates in solution (breaking MB—H_2_O and Fe_3_O_4_/BC—H_2_O H-bonds) and displacing the water molecules from the adsorbent surface (e.g., causing randomness in the system) when increasing the temperature value [24,53]. Positive ΔS° values are also likely to account for different oxygenated functionalities (-OH and -C=O) on the Fe_3_O_4_/BC surface in water solutions. These functionalities participate in the interaction process with adsorbate molecules via either electrostatic (-COO—MB^+^/-C-O^−^—MB^+^) or H bond (-COOH—MB/-OH—MB) interactions (i.e., the randomness of the adsorption process at the solid–liquid interface). These interactions were explained by FTIR analysis of Fe_3_O_4_/BC after MB adsorption, as explained in Section 3.6.

Thermodynamically, the negative ∆G values (Table 1; Figure 10b) can also be used to judge the effectiveness of electrostatic interactions between MB and Fe_3_O_4_/BC. Herein, the negative ΔG° values were observed for MB adsorption at all tested concentrations, indicating the interaction of MB with Fe_3_O_4_/BC [24]. Also, the negative values of ΔG° were slightly increased with increased temperature at all tested concentrations of MB, while the ΔG° values greatly declined with increasing initial concentrations of MB adsorbate (Table 1). These findings indicate the low contribution of electrostatic interactions in the adsorption process (in particular at low concentrations) along with the favorability of adsorption at higher temperatures. At higher temperatures, the conversion of MB liquid to solid forms should be able to take place spontaneously through the interaction of MB with the protonated/deprotonated Fe_3_O_4_/BC adsorbent functionalities as follows.

ΔG° (adsorption) for MB = ΔG°_Fe3O4/BC_ + ΔG°_Fe3O4/BC-MB_ + ΔG°_Fe3O4-MB_ + ΔG°_BC-MB_ (14)

### 3.4. Isotherm Studies of MB Adsorption

Herein, adsorption equilibrium data of MB on Fe_3_O_4_/BC at three temperature levels (27, 35, and 45 °C) were described using Langmuir (Figure 10c), Freundlich (Figure 10d), and Temkin (Figure 10e) models to understand adsorption mechanism.

From Table 2 and Figure 10c–f, it is evident that the Freundlich model presents a satisfactory fit for the MB equilibrium adsorption data onto Fe_3_O_4_/BC at all temperature ranges, with a high correlation factor (R^2^) of 0.99 (for linear plot; Figure 10d) and the lowest standard error (ARE; for simulation graph; Figure 10f). Notably, the Langmuir isotherm cannot be used to explain the adsorption data in this study due to the poor fitting with the experimental data of MB dye on Fe_3_O_4_/BC (Figure 10c,f). These findings indicated that the uptake mechanism of MB molecules appeared to follow multilayer adsorption on Fe_3_O_4_/BC surfaces that possess active surface sites with different energies (i.e., heterogeneous adsorption sites). This observation was supported by high theoretical adsorption intensity (*n*) values in a range of 1.8–1.5 for MB (Table 2), verifying the favorable adsorption onto heterogeneous adsorbent surfaces [20].

Based on Temkin constants (Table 2), the adsorption process between the MB molecules and Fe_3_O_4_/BC surface (physical or chemical interactions) can be interpreted theoretically. As seen, the low values of the heat of adsorption parameter (b_T_, kJ mol^−1^: 0.34–0.30) indicate the dominant role of physisorption interactions between the MB and Fe_3_O_4_/BC surface sites [54].

From the Freundlich isotherm, the values of the *k_F_* constant were increased from 18.4 to 28.2 mg^(1−n)^ L^n^ g^−1^ for MB as the temperature increased from 27 to 45 °C. This indicates that Fe_3_O_4_/BC has a good adsorption affinity for MB dye and that adsorption affinity may increase with temperature (i.e., improved interactions between the MB and Fe_3_O_4_/BC at higher temperatures). However, it should be noted that the estimated values of Temkin model parameters (*A_T_* (binding energy) and *b_T_* (heat of adsorption)) have been slightly affected by the alteration of adsorption temperature, suggesting the slight contribution of temperature to the MB adsorption on the Fe_3_O_4_/BC surface.

### 3.5. Kinetics Studies of MB Adsorption

**Figure 11:**
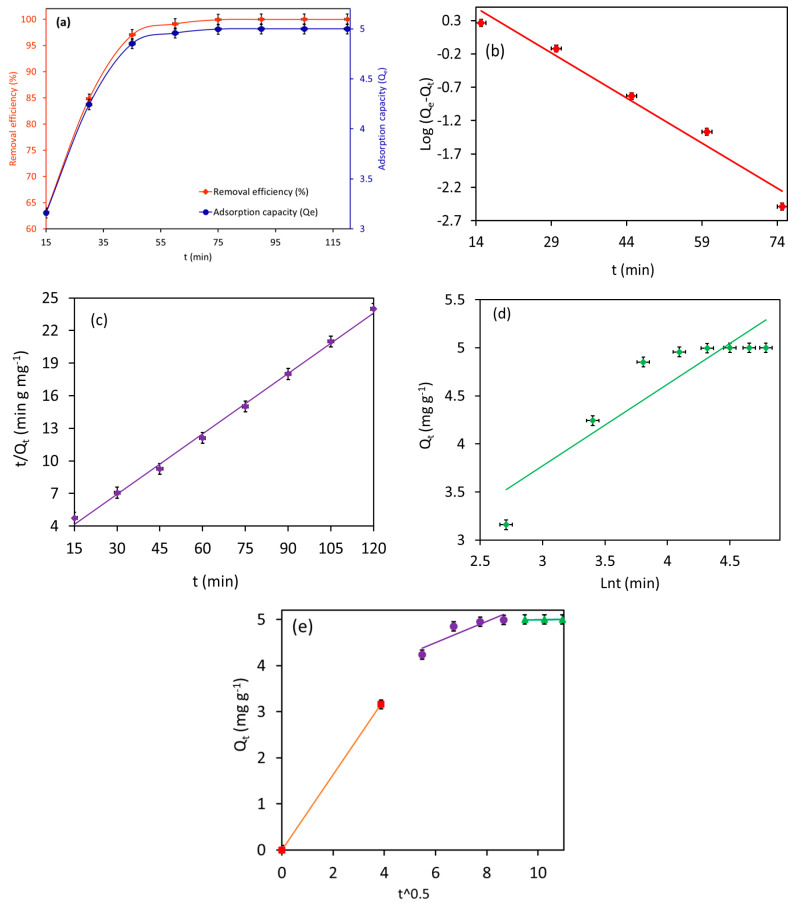
Plots of (**a**) effect of contact time on MB adsorption, (**b**) PFO kinetic, (**c**) PSO kinetic, (**d**) Elovich kinetic, and (**e**) intraparticle diffusion model for MB adsorption onto the Fe_3_O_4_/BC (at pH = 7, adsorption dose = 2.0 g L^−1^, MB initial concentration = 10 mg L^−1^, and temperature 27 °C).

Figure 11a illustrates the adsorption rate of 10.0 mg L^−1^ MB by Fe_3_O_4_/BC (2.0 g L^−1^) as a function of contact time (15–120 min) in fixed conditions (temperature of 27 °C and pH 7).

Notably, the Fe_3_O_4_/BC showed faster uptake for MB dye molecules (in 45 min, Figure 11a), confirming the high affinity of the developed sorbent for organic dye removal. The obtained time-dependent adsorption data of MB on Fe_3_O_4_/BC were also examined with the help of various kinetic models (PFO, PSO, Elovich, and IPD models) to understand the rate-controlling adsorption step. The kinetic plots of PFO, PSO, and Elovich models and their fitting to the experimental data are illustrated in Figure 11b–e, while the computed constants for these kinetic models are summarized in Table 3.

As shown in Table 3(a), the adsorption of MB onto Fe_3_O_4_/BC is better fitted to the PSO model (R^2^ of 0.99) than PFO (R^2^ of 0.97) and Elovich models (R^2^ of 0.83). The good agreement between the experimental and calculated adsorption capacity was shown by the PSOM (with ∆*Q* = 0.40) compared to PFOM (with ∆*Q* = 8.1). This finding suggests that the adsorption of MB onto Fe_3_O_4_/BC is likely to be controlled by chemisorption (electrostatic) interaction. It is also apparent that the prepared Fe_3_O_4_/BC adsorbent exhibited an uptake rate for MB (k_2_ = 0.025 g mg^−1^ min^−1^). Similar results were reported by Elkady et al. [29].

Notably, the Elovich plot has a low R^2^ (0.83) value which does not support the fact that the Fe_3_O_4_/BC adsorbed the MB pollutants through pure chemisorption (i.e., some form of physical absorption is also responsible for the current MB absorption). From Table 3(a), the higher value of α (3.6 mg g^−1^ min^−1^) than the value of β (1.2 mg g^−1^) suggested that the rate of MB adsorption onto Fe_3_O_4_/BC was much higher than the rate of their desorption at equilibrium [20]. Such results can be explained by the interactions of surface functionalities of Fe_3_O_4_/BC with adsorbate molecules via electrostatic (-COO—MB+/-C-O-—MB+) and non-electrostatic (e.g., H bond) interactions.

Referring to the IPD plots for MB adsorption (Figure 11e), it is quite clear that the adsorption of MB onto the Fe_3_O_4_/BC surface followed the three-step process at the given initial MB concentration. From the IPD plots (Figure 11e), the first, very steep stage (0–15 min for MB) reflected the instantaneous adsorption on the external adsorbent surface due to mass transfer. The second even steeper stage suggested MB adsorption due to boundary layer formation (film diffusion) [29]. The third gradual stage (linear portion) is attributed to the adsorption region in which the intraparticle diffusion is the rate-limiting step. It is evident from Figure 11e that the linear lines of IPD plots did not pass through the origin, suggesting that the rate-limiting adsorption mechanism was not only the intraparticle diffusion process [29]. In Table 3(b), the larger value of the intercept (boundary layer thickness, C_2_) and smaller value of the constant (Kd_2_) of IPD for the adsorption of MB on Fe_3_O_4_/BC indicate the high surface affinity (fast adsorption rate) for interaction with MB molecules (at the second stage) compared to that of their intraparticle diffusion (third stage) [29]. Based on these observations, intraparticle diffusion is the major rate-determining (slow) step for the adsorption of MB pollutants on the prepared Fe_3_O_4_/BC nanobioadsorbent [29].

These kinetic models may be evidence for the physicochemical adsorption process for MB adsorption onto the Fe_3_O_4_/BC along with the electrostatic/non-electrostatic interaction between the functional sites of Fe_3_O_4_/BC and MB (Figure 9) [15], which was further confirmed by comparative FTIR analysis before and after the adsorption process in Section 3.1.

### 3.6. Adsorption Activation Energy

**Figure 12:**
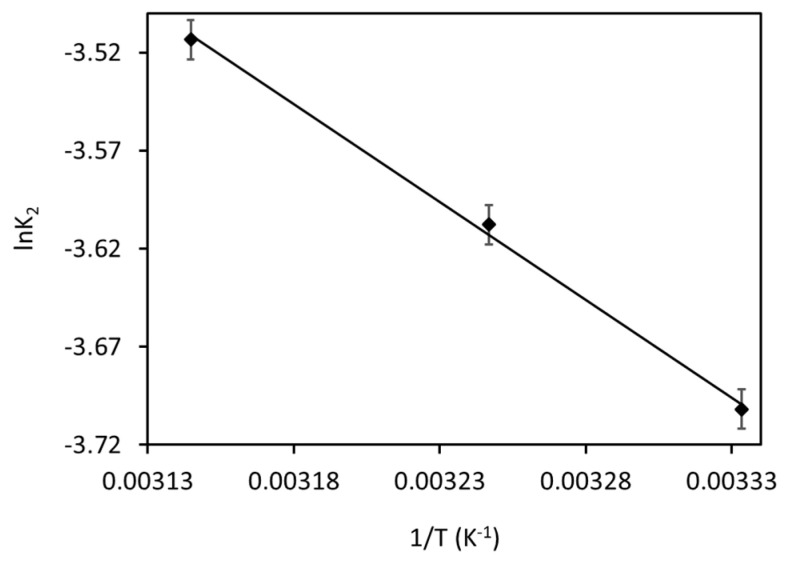
Arrhenius plot for the MB adsorption onto Fe_3_O_4_/BC.

To find the Ea, a graph made between lnK_2_ and 1/T is shown in Figure 12. The Ea value for current MB adsorption was found to be 8.3 kJ mol^−1^, which confirms that the present MB adsorption is majorly governed by physisorption. This result can also be confirmed through previous studies [26].

### 3.7. Adsorption Mechanism, Reusability Features, and Economic Efficiency

#### 3.7.1. FTIR Analysis for MB-Loaded Fe_3_O_4_/BC: Adsorption Mechanism

The electrostatic/H-bonding interactions of MB dye with functional surface sites of Fe_3_O_4_/BC were confirmed by FTIR analyses before and after adsorption, as represented in Figure 1. The FTIR spectrum of Fe_3_O_4_/BC after MB adsorption in Figure 1 (green line) and the red-shift in the -OH stretching frequency from 3412 cm^−1^ (before adsorption) to 3344 cm^−1^ (after adsorption) may result from the H-bond formation between cationic MB and hydroxyl Fe_3_O_4_/BC. The carbonyl peak of amide I/OH bending and N-H stretching amide II bands also show a larger shift towards the lower wavenumber (for amide I/OH bending; 1636 cm^−1^ (before adsorption) to 1600 cm^−1^ (after adsorption)) and disappearance ((for amide II; 1543 cm^−1^ (before adsorption)), assuming a strong electrostatic interaction of MB with amide functional sites. Similarly, the disappearance in vibrational peaks (1404 cm^−1^ and 1075 cm^−1^) (Figure 1, red line) was obtained due to electrostatic interaction between MB and functional sites of Fe_3_O_4_/BC (Figure 1, green line). The existence of the C-S stretching bond of MB molecules was further demonstrated by characteristic peaks at 1108 cm^−1^ (after adsorption) that supported the idea that MB was adsorbed on Fe_3_O_4_/BC. These findings indicate that there was a specific electrostatic interaction between Fe_3_O_4_/BC and MB (Figure 9).

#### 3.7.2. Comparative Performance Based on the Partition Coefficient Metric

The performance of Fe_3_O_4_/BC relative to the reported adsorbent in the literature for MB dye removal was evaluated through a comparative analysis based on maximum capacity (*Q_o_* mg g^−1^), removal efficiency (%), and partition coefficient (PC: L g^−1^). In this study, PC (L g^−1^) performance data have been considered a feasible key metric for comparative analysis because the operating conditions can alter the removal efficiency (%) and *Q_o_* values (e.g., initial concentration levels of the target compounds and mass of loaded adsorbent). In this regard, PC equations (Equation (15) or Equation (16)) were developed to provide a more reliable judgment on the performance by avoiding the variation in experimental conditions by considering the initial or residual pollutant concentrations and loaded mass of adsorbent in the capacity form [20].

(15) $$PC=Adsorption\;capacity\;(Q_{e})/Final\;concentration\;(C_{e})$$

(16) $$PC=Adsorption\;capacity\;(Q_{e})/(Initial\;concentration\;\left(C_{o}\right)\;*\;Removal\;rate\;\left(Q_{t}\right))$$

**Figure 13:**
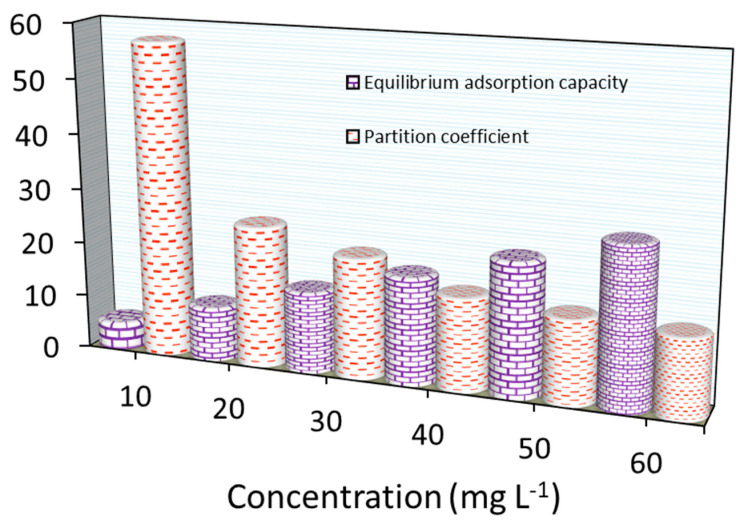
Partition coefficient and equilibrium adsorption capacity for MB adsorption onto Fe_3_O_4_/BC at various concentrations.

As summarized in Figure 13, the PC value (in g L^−1^) for MB adsorption decreased when increasing the initial loading concentrations. The PC values were ~57.0 to ~14.0 L g^−1^ for Fe_3_O_4_/BC as the MB concentration changed from 10.0–60.0 mg L^−1^. If these results are compared with those measured previously (for BC-based adsorbents) (as seen in Table 4), Fe_3_O_4_/BC has displayed the highest PC with noticeable *Q_o_* values. Based on PC values, Fe_3_O_4_/BC appears to be one of the most effective treatments for these contaminants. This feature may suggest the high efficiency of Fe_3_O_4_/BC for MB removal from groundwater reservoirs.

#### 3.7.3. Economic Efficiency, Reusability Features, and Implementation of Fe_3_O_4_/BC

**Figure 14:**
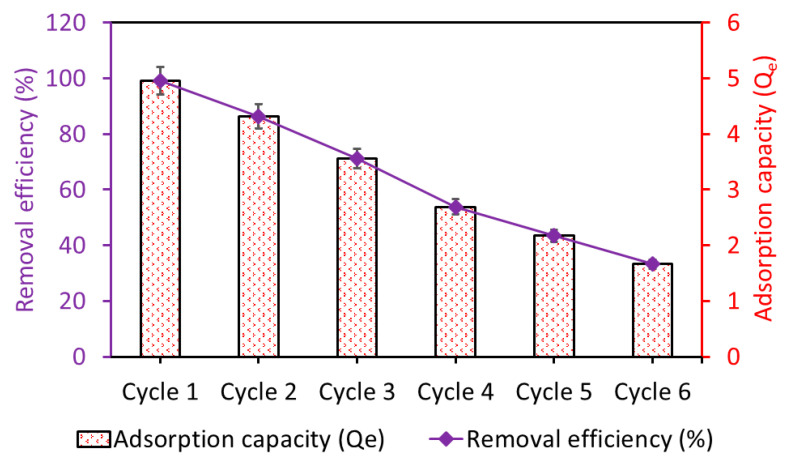
Adsorption stability of the regenerated Fe_3_O_4_/BC bioadsorbent for MB pollutant.

The reusability of Fe_3_O_4_/BC was investigated by running up to six cycles of adsorption–desorption, as shown in Figure 14. The prepared Fe_3_O_4_/BC bioadsorbent demonstrated excellent stability over the first two cycles, followed by a gradual decline in adsorption performance to achieve a total reduction in performance of 33% for MB after the sixth reusability cycle (Figure 14).

Being a multifunctional material, the easily prepared Fe_3_O_4_/BC can be a useful nanobiosorbent for wastewater treatment because of its low production cost (around 0.07 USD/g) (computed manually), which is comparatively lower than that of those reported previously such as Fe_2_O_3_ (1.2 USD/g), Fe_3_O_4_ (0.44 USD/g), TiO_2_ (0.18 USD/g), ZVNI (0.14 USD/g), Fe_2_O_3_-ZrO_2_/BC (0.10 USD/g), Fe_2_O_3_-SnO_2_/BC (0.07 USD/g), and various other adsorbents [21]. The low application cost of magnetic Fe_3_O_4_/BC is mainly attributed to the high mass yield production from cheap and readily available resources of carbon frameworks (BC seeds), good performance stability over six reusable cycles, and the ease of recovery of exhausted sorbent from solution using an external magnetic field (low consumption of energy for recovery). The findings from this study indicate that Fe_3_O_4_/BC is an environmentally friendly and cost-effective adsorbent with significant adsorption capabilities for both inorganic and organic pollutants in wastewater treatment.

## 4. Conclusions

The magnetic nanobiomaterial Fe_3_O_4_/BC was synthesized for environmental applications via the incorporation of iron oxide in a carbon framework derived from cumin seeds through the co-precipitation method. The reported nanobiomaterial is eco-friendly, cost-effective, magnetic, and highly functionalized. This nanobiomaterial exhibited remarkable efficacy in extracting MB from water. Adsorption isotherms were also found to obey Freundlich models, indicating possible multilayer adsorption onto heterogeneous active adsorption sites. The current nanobiomaterial demonstrated superior efficiency such as maximal adsorption efficacy and partition coefficient compared to existing materials. Here, 2.0 g L^−1^ of Fe_3_O_4_/BC removed ~99% of MB with a 10 mg L^−1^ concentration at pH = 7, temperature = 27 °C, and contact time = 60 min. In contrast, its maximum equilibrium adsorption capacity (5.0 mg g^−1^) and maximum partition coefficient (57.0 L g^−1^) were observed at the starting concentration (10.0 mg L^−1^, 31.0 μM). Fe_3_O_4_/BC showed a better partition coefficient than previously reported BC seeds (acid-washed) and other BC-based adsorbents. The enhanced adsorption efficiency of the present nanobiomaterial is attributed to the excessive functional groups on its surface, as confirmed by the FTIR study. The utilized adsorbent also showed a high regeneration ability and could be reutilized over multiple adsorption–desorption cycles without a large reduction in adsorption capacity. Given the potential for the future, additional research including a range of contaminants will likely be required to enhance the effectiveness and efficiency of these materials for a variety of water treatment applications. In the future, the antibacterial activities of these materials should also be addressed to assess the antibiofilm nature of adsorbents.

## Figures and Tables

**Table 1:** Thermodynamic parameters for MB adsorption at various concentrations *.

Order	C_o_ mg g^−1^	∆H kJ mol^−1^	∆S kJ mol^−1^ K^−1^	∆G kJ mol^−1^		
				300 K	308 K	318 K
1.	10	+12.6	+0.07	−10.1	−10.5	−10.8
2.	20	+23.9	+0.10	−8.1	−8.8	−9.4
3.	30	+26.0	+0.11	−7.7	−8.4	−9.2
4.	40	+33.8	+0.13	−7.0	−8.1	−9.0
5.	50	+31.5	+0.13	−6.8	−7.4	−8.5
6.	60	+29.7	+0.12	−6.6	−7.4	−8.3

* Experimental conditions: pH = 7, adsorption dose = 2.0 g L^−1^, and contact time = 120 min.

**Table 2:** The isothermal parameters for the adsorption of MB onto Fe_3_O_4_/BC *.

Order	Temp. °C	Langmuir					Freundlich				Temkin			
		*Q*_o_ mg g^−1^	b L mg^−1^	R_L_	R^2^	ARE	k_F_ mg^(1−n)^L^n^ g^−1^	n	R^2^	ARE	A_T_ L g^−1^	b_T_ kJ mol^−1^	R^2^	ARE
1.	27	40.0	1.0	0.09	0.90	13.1	18.4	1.8	0.99	4.3	15.5	0.34	0.90	21.8
2.	35	42.0	1.2	0.07	0.90	11.7	21.7	1.7	0.99	4.3	18.3	0.33	0.90	20.9
3.	45	48.5	1.3	0.07	0.91	8.5	28.2	1.5	0.99	3.2	19.7	0.30	0.92	20.4

* Experimental conditions: pH = 7, adsorption dose = 2.0 g L^−1^, MB initial concentration = 10–60 mg L^−1^, and contact time = 120 min.

**Table 3:** (a) Parameters of the PFO, PSO, and Elovich kinetic models for the adsorption of MB onto Fe_3_O_4_/BC *. (b) Parameters of the Weber–Morris model for adsorption of MB onto Fe_3_O_4_/BC *.

**(a)**											
**Order**	**PFO Kinetics** ***Q*_exp_ (mg g^−1^) = 5.0**				**PSO Kinetics** ***Q*_exp_ (mg g^−1^) = 5.0**				**Elovich**		
	***Q*_cal_** **mg g^−1^**	**∆*Q***	**K_1_**	**R^2^**	***Q*_cal_** **mg g^−1^**	**∆*Q***	**K_2_**	**R^2^**	**α**	**β**	**R^2^**
1.	13.1	8.1	0.103	0.97	5.4	0.40	0.025	0.99	3.6	1.2	0.83
**(b)**											
**Order**	**Weber–Morris Model**										
	**Second Stage** **(Film Diffusion)**					**Third Stage** **(Intraparticle Diffusion)**					
	**Kd_1_**		**C_1_**		**R^2^**	**Kd_2_**		**C_2_**		**R^2^**	
1.	0.231		3.1		0.80	5 × 10^−5^		5.0		0.99	

* Experimental conditions: pH = 7, adsorption dose = 2.0 g L^−1^, MB initial concentration = 10 mg L^−1^, contact time = 120 min, and temperature 27 °C.

**Table 4:** Summary of experimental data obtained for performance evaluation of adsorbents used to remove MB from water and comparative Langmuir maximum adsorption capacity (*Q_o_*) values of adsorbents *.

Order	Adsorbent	Final Concentration, C_e_ (μM)	Time, Min	Adsorbent Dose, g L^−1^	*Q_e_* (C_o_ − C_e_/m), mg g^−1^	*Q_o_*, mg g^−1^	PC, L g^−1^	Ref.
1.	rGO-BC@ZrO_2_	~0.3	90	2.0	~4.95	~23.4	51.4	[19]
2.	Fe_2_O_3_-ZrO_2_/BC	~0.4	-	3.0	~3.31	~38.1	55.2	[20]
3.	Fe_3_O_4_-SnO_2_/BC	~0.64	90	2.0	~4.90	~58.8	23.9	[21]
4.	MnFe_2_O_4_/BC	~0.20	45	3.0	~3.31	~10.1	52.6	[39]
5.	Acid-washed BC seeds	~0.59	60	1.0	~9.81	~73.5	51.6	[55]
6.	Fe_3_O_4_/BC	~0.27	60	2.0	~4.96	~40.0	57.2	This study

* Experimental conditions: pH = 7, MB initial concentration = 10 mg L^−1^ (~31.0 μM), and temperature = 27 °C.

## Data Availability

The original contributions presented in this study are included in the article and Supplementary Materials. Further inquiries can be directed to the corresponding authors.

## Supplementary Materials

The following supporting information can be downloaded at: https://www.mdpi.com/article/10.3390/ma18092049/s1, Figure S1: FTIR spectrum of BC. Reprinted from our previous study with permission from Siddiqui and Chaudhry [39], Copyright (2018) Elsevier (License No. 5990980697967).
