# Supplementary material for: Fe3O4/BC for Methylene Blue Removal from Water: Optimization, Thermodynamic, Isotherm, and Kinetic Studies
Source: Materials (Basel). 2025 Apr 30;18(9):2049. doi: 10.3390/ma18092049 (PMC12072255; doi:10.3390/ma18092049)
Supplement: Supplementary file 1 [file materials-18-02049-s001.zip › materials-3522937-supplementary.pdf]

materials

MDPI

Supplementary Data

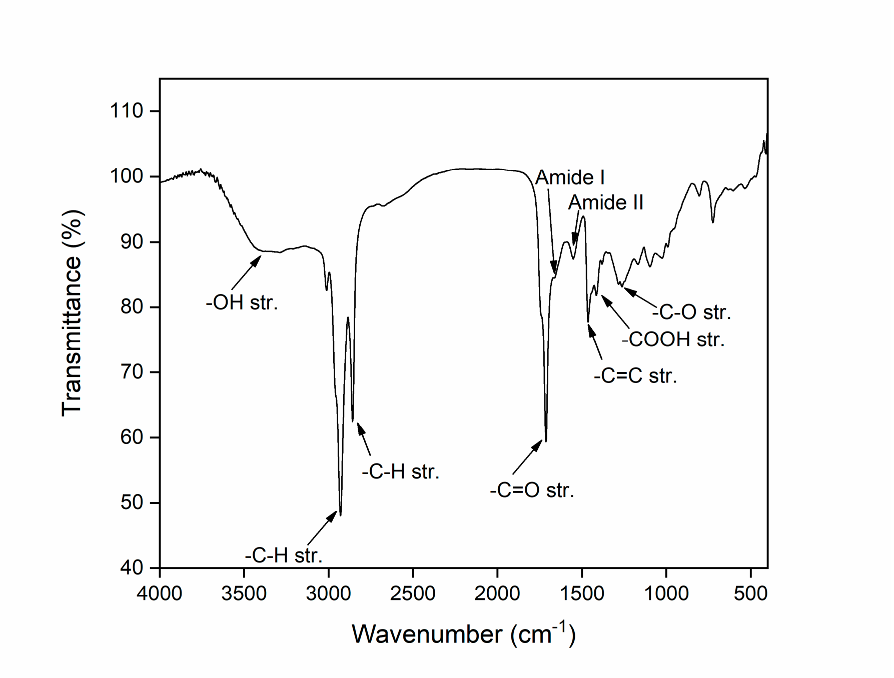

Figure S1. FTIR spectrum of BC*. Reprinted from our previous study with permission from Siddiqui and Chaudhry [1], Copyright (2018) Elsevier (License No. 5990980697967).

Reference

1. Siddiqui, S. I., & Chaudhry, S. A. (2018). Nigella sativa plant-based nanocomposite-MnFe2O4/BC: An antibacterial material for water purification. *Journal of cleaner production*, *200*, 996-1008.

*This study is an extension of our previous studies [1], therefore, we obtained the copyright permission from the publisher.
